# Molecular analysis of cox-1 and 18S rRNA gene fragments of *Eimeria* species isolated from endangered grouse: capercaillie (*Tetrao urogallus*) and black grouse (*Tetrao tetrix*)

**DOI:** 10.1007/s00436-018-6171-5

**Published:** 2018-12-18

**Authors:** Tomasz Stenzel, Daria Dziewulska, Maria Michalczyk, Dorota B. Ławreszuk, Andrzej Koncicki

**Affiliations:** 1 Department of Poultry Diseases, Faculty of Veterinary Medicine, University of Warmia and Mazury in Olsztyn, ul. Oczapowskiego 13, 10-719 Olsztyn, Poland; 2 Department of Parasitology and Invasive Diseases, Faculty of Veterinary Medicine, University of Warmia and Mazury in Olsztyn, ul. Oczapowskiego 13, 10-719 Olsztyn, Poland; 3 Institute of Biology, University of Białystok, ul. Konstantego Ciołkowskiego 1J, 15-245 Białystok, Poland

**Keywords:** Black grouse, Capercaillie, Cox-1 gene, Genetic diversity, Phylogenetic analysis, 18S rRNA gene

## Abstract

This paper is the first record describing the molecular analysis of *Eimeria* species occurring in capercaillie (*Tetrao urogallus*) and black grouse (*Tetrao tetrix*) which inhabit northern Eurasia and are species critically endangered of extinction. Actions undertaken to protect endangered species, such as breeding individuals in closed aviaries, could allow saving those birds, but they also pose risk of accidental healing of invasive diseases, like coccidiosis. Therefore, an investigation was conducted on fecal samples collected from the capercaillies and black grouse originating from the Kirov region (Russia) and breeding centers located in Poland. Results indicate that the average prevalence of *Eimeria* revealed 72% (average OPG = 3548) and 80% (average OPG = 5220) in capercaillies and black grouse respectively. Most of the *Eimeria* spp*.* oocysts were non-sporulated; however, two different morphological types were observed. The phylogenetic analysis of cox-1 and 18S rRNA genes revealed the analyzed *Eimeria* sequences to belong to two species. In addition, it showed some similarities between both analyzed genes. Most of the sequences obtained from both grouse species coccidia belonged to one species partially homologous to the *Eimeria* spp*.* isolated from ring-necked pheasant (approx. 94 and 96% for cox-1 and 18S rRNA genes, respectively). Two strains isolated from capercaillies imported from Russia were related to turkey coccidia: *E. innocua* and *E. dispersa* (97–99% homology) in the cox-1 gene analysis and only one of them was related to those *Eimeria* species in the 18S rRNA gene analysis (98–99% homology).

## Introduction

Capercaillie (*Tetrao urogallus*) and black grouse (*Tetrao tetrix*) are large grouse birds that belong to the *Galliformes* order. They are boreal species breeding across the northern Eurasia. Since the mid-twentieth century, these two species have been facing a dramatic decrease in their numbers in Western Europe and in Poland. They are entered into the “Polish Red Book of Animals”—the capercaillie as a “critically endangered” species (CR category) and the black grouse as an “endangered” one (EN category) (Głowaciński 2001). The observed rapid decline in their numbers is caused by the loss of optimal habitats or forage base, excessive predatory pressure, geographical isolation, and depletion of the gene pool and anthropogenic factors (tourism, physical barriers such as forest planting fences, etc.) (Moreno-Opo et al. 2015; Thiel et al. 2011). The small genetic pool on the one hand leads to the inbred depression but can also predispose to parasitic invasions, which have been reported in the capercaillie (Isomursu et al. 2012). The response to this situation assumes implementation of numerous programs for active protection of the capercaillie and black grouse across Europe (Rutkowski et al. 2017; Siano and Klaus 2013; Strzała et al. 2015) to halt further loss of their number, enlarge their populations, and assure their long-term protection in Europe. These programs include releasing into the forest individuals from domestic breeding centers and birds translocated from the wild population from various geographical regions. The above actions undertaken to protect endangered species could allow for saving the capercaillie and black grouse. On the other hand, they pose risk of accidental healing of infectious/invasive diseases or of introducing new infectious agents or their genetic variants to the new geographical locations. Keeping birds in unnatural conditions, like a closed breeding aviary, and high animal density also foster invasive diseases, especially with parasites of the simple life cycle. This phenomenon was also observed in the case of coccidia (Clark et al. 2017).

There are reports of coccidiosis infections in wildlife black grouse and capercaillie (Jankovska et al. 2012; Millán et al. 2008; Obeso et al. 2000). However, none of the coccidia that parasite on those species have been studied with the use of molecular methods. For the above reason, the aim of this study was to evaluate the genetic diversity of *Eimeria* (*E*.) species parasites isolated from fecal samples of capercaillies and black grouse originating from free-ranging breeding centers located in Poland and these transported from the Kirov region (Russia).

## Materials and methods

### Ethical statement

This study was carried out on fecal samples collected from the transport cages and aviaries, and no manipulations of live animals were done. The grouse were transported and kept as a part of the EU Project LIFE “Active protection of lowland populations of Capercaillie in the Bory Dolnośląskie and Augustowska Primeval Forest” according to permission no. DZP-WG.6401.03.100.2016.km (release date 31 May 2016) issued by General Director of Environment Protection.

### Fecal samples

In September 2016, 24 capercaillies (age not determined) were transported into the Augustowska Primeval Forest from the Kirov region (Russia) (translocation distance 1525 km). Samples of their feces were collected from each transport cage immediately after the birds had been released. In October 2016, fecal samples were collected in aviaries of breeding centers inhabited by adult birds (*n* = 15). Additionally in August 2017, 18 samples were collected from young birds (3–4 months old) placed in adaptive aviaries and prepared for reintroduction with the “born to be free” method (Merta et al. 2015). Those capercaillies originated from the breeding center located in the Leżajsk State Forestry District (Southern East of Poland). The total number of examined samples was 57.

Fecal samples of adult black grouse (*n* = 10) were sent by the Jedwabno State Forestry District to Department of the Poultry Diseases, Faculty of Veterinary Medicine, University of Warmia and Mazury in Olsztyn (Poland) in June 2017.

### Parasitological screening

The collected fecal samples were analyzed by flotation in a Darling fluid (a solution of saturated sodium chloride and glycerol in a ratio of 1:1). They were mixed on the mesh placed in a Petri dish. The resultant slurry was centrifuged at 800×*g* for 5 min. To determine the number of oocysts, coccidia were counted in a McMaster slide (OPG). The fecal samples containing oocysts were placed in a 2% (*w*/*v*) potassium dichromate solution, poured into Petri dishes, and kept at room temperature in the dark for sporulation.

### DNA extraction

One hundred-microliter aliquots of supernatant from all centrifuged samples containing oocysts were transferred into a microcentrifuge tube containing 1400 μL of 0.9% sodium chloride and centrifuged again at 2000×*g* for 5 min. To damage the oocyst’s wall, the resultant sediment was resuspended in 500 μL of ATL lysis buffer (Qiagen, Germany). Next, the steel bead was added into each tube and tubes were placed in Tissuelyser II (Qiagen, Germany) homogenizer for 2 min with extensive shaking (30x/1 s). After one step of 1 min incubation on ice, the samples were placed in Tissuelyser II again (the same conditions). Next, they were centrifuged at 7000×*g* for 1 min. The following steps of DNA extraction were conducted with a DNeasy mini kit (Qiagen, Germany) in accordance with manufacturer instructions.

DNA concentration and purity was estimated using a NanoDrop 2000 (Thermo Scientific, USA) spectrophotometer, and the samples were stored at − 80 °C until further analysis.

### PCR amplification and sequencing

Products of approximately 800 bp of the mitochondrial cytochrome oxidase subunit 1 (cox-1) and approximately 1320 bp of 18S rRNA gene were amplified with the primers designed by Miska et al. (2010) and Yang et al. (2012). Both PCRs were performed using a HotStarTaq Plus Master Mix (Qiagen, Germany) in a MasterCycler Pro thermocycler (Eppendorf, Germany). The composition of the reaction mixture used in both assays was as follows: 10 μL of HotStarTaq Master Mix, 0.1 μL of each primer (concentration 100 μM), 2 μL of Coralload loading dye, and 2 μL of template DNA. The reaction mixture was topped up to the volume of 20 μL with ribonuclease-free water. The cox-1 gene fragment was amplified under the following conditions: initial denaturation at 95 °C for 5 min followed by 34 denaturation cycles at 94 °C for 30 s, annealing at 55 °C for 30 s, and chain elongation at 72 °C for 1 min, whereas both steps of hemi-nested PCR amplification of the 18S rRNA gene were conducted under the following conditions: initial denaturation at 95 °C for 5 min followed by 45 denaturation cycles at 94 °C for 30 s, annealing at 55 °C for 30 s, and chain elongation at 72 °C for 2 min. The final elongation after the last cycle in both assays was performed at 72 °C for 10 min. The amplification products were used for agarose gel electrophoresis whose results were read using the GelDoc XR+ gel imaging system (Bio-Rad, Italy).

All of the amplified gene fragments were purified with a Concentrate Clean-Up kit (A&A Biotechnology, Poland) and sequenced using Sangers method in Genomed S.A. (Poland).

### Phylogenetic analysis

The resulting sequences were assembled in the SeqMan Pro application (DNASTAR, USA) and checked manually for sequencing errors using the same software. Sequences of the cox-1 and the 18S rRNA genes were compared with respectively 20 and 17 sequences of these genes of *Eimeria* species obtained from gallinaceous birds. The sequences used for comparison and their GenBank accession numbers are listed in Table 1. The obtained in this study and listed in GenBank sequences were aligned using the Clustal W and Clustal V methods and MegAlign software (DNASTAR, USA). All aligned sequences were trimmed at the same 5′ and 3′ ends and used for further analyses. Nucleotide and amino-acid identity matrices were generated with MegAlign Pro software (DNASTAR, USA). Phylogenetic trees were generated with the maximum likelihood method (Felsenstein 1981) in Mega 7 software (Kumar et al. 2016). Bootstrap values were calculated for 1000 replicates of the alignment. Gaps in the alignment were treated as missing characters. The phylogenetic trees were rooted with the sequence of *Toxoplasma gondii* (GenBank accession no. KM657810 and L37415).

**Table 1:** The sequences of cox-1 and 18 s rRNA genes of different *Eimeria* species used to perform phylogenetic analysis

Scientific name	Accession number	
	Cox-1	18 s rRNA
*Eimeria (E.) acervulina*	FJ236428	KT184333
*E. adenoides*	FR846201; FR846202	KC305172
*E. brunetti*	HM771675	U67116
*E. dispersa*	HG793048	HG793041
*E. gallopavonis*	HG793051	KT184344
*E. innocua*	HG793049	HG793045
*E. irresidua*	JQ993694	HQ173832
*E. maxima*	FJ236459	DQ538348
*E. meleagridis*	HG793046	HG793040
*E. meleagrimitis*	HG793050	HG793044
*E. mitis*	FR796699	FR775302
*Eimeria sp. Meleagris gallopavo*	HM117018	HM117016
*E. nectrix*	EU025108	DQ136185
*E. pavonina*	JN596590	JN596589
*Eimeria sp. Perdix perdix*	KJ547709	–
*Eimeria sp. Phasianus colchicus*	HM117019; KJ547708	HM117008
*E. praecox*	KT184378	FJ236371
*E. tenella*	HM771676	EF210326

### Accession numbers

Sequences of *Eimeria* spp. isolates obtained in this study were submitted to the GenBank database to generate accession numbers (MG595956-MG595963 and MG825660-MG825666).

## Results

### Oocysts in fecal samples

All samples obtained from the capercaillies imported from the Kirov region were positive for *Eimeria* spp*.* oocysts, whereas the prevalence of *Eimeria* parasites in the samples collected from birds located in Augustowska Primeval Forest and from Leżajsk State Forestry District was at 33 and 66%, respectively. The average prevalence of *Eimeria* in the examined capercaillies oscillated at 72%. The average OPG was 3548 (233–19,436). Eighty percent of the samples collected from black grouse were positive for *Eimeria* spp*.* oocysts with average OPG at 5220 (400–100,000).

**Fig. 1:**
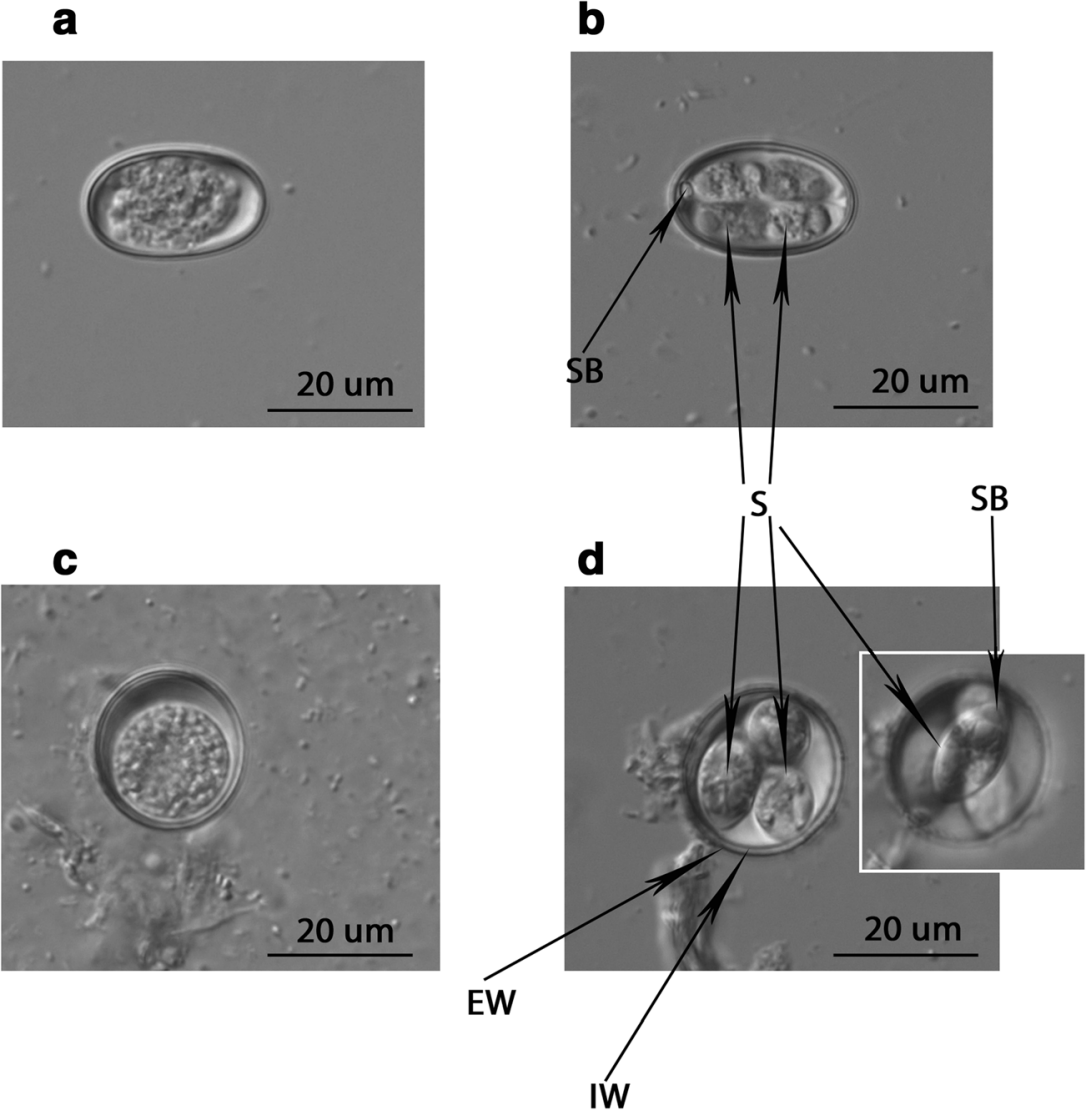
*Eimeria* spp*.* oocysts isolated from investigated grouse species. **a**, **c** Non-sporulated oocysts. **b**, **d** Sporulated oocyst. Abbreviations: EW external wall, IW internal wall, S sporocyst, SB Stieda body

Two different morphological types of oocysts (ellipsoid and round in shape) were observed. Both sporulating morphotypes were characterized by a smooth 0.6 μm-thick bi-layered wall. The oocysts measured 24.6 × 12.6 μm and 19.4 × 17.7 μm, and their length/width (L/W) ratio was 1/0.51 and 1/0.91, an average for the ellipsoid and round morphotypes, respectively. Oocyst micropyle and polar granule were absent. The oocysts contained four roughly ovoid sporocysts measuring 8.6 × 4.5 and 11.4 × 7. 4 μm with a sporocyst L/W ratio of 1/0.53 and 1/0.64 for the ellipsoid and round morphotype, respectively (Fig. 1). The sporulation time reached 36 and 48 h for the ellipsoid and round morphotype, respectively. From all the samples positive in parasitological screening, only 8 (4 out of each grouse species) were positive in the cox-1 gene assay and 7 (3 from black grouse and 4 from capercaillie) in the 18S rRNA assay.

All cox-1 gene and 18S rRNA gene amplicons were successfully sequenced and assembled into contigs (assembled sequences with the length of 690–786 and 1313–1404 nucleotides for cox-1 and 18S rRNA genes, respectively).

### Phylogenetic analysis

**Fig. 2:**
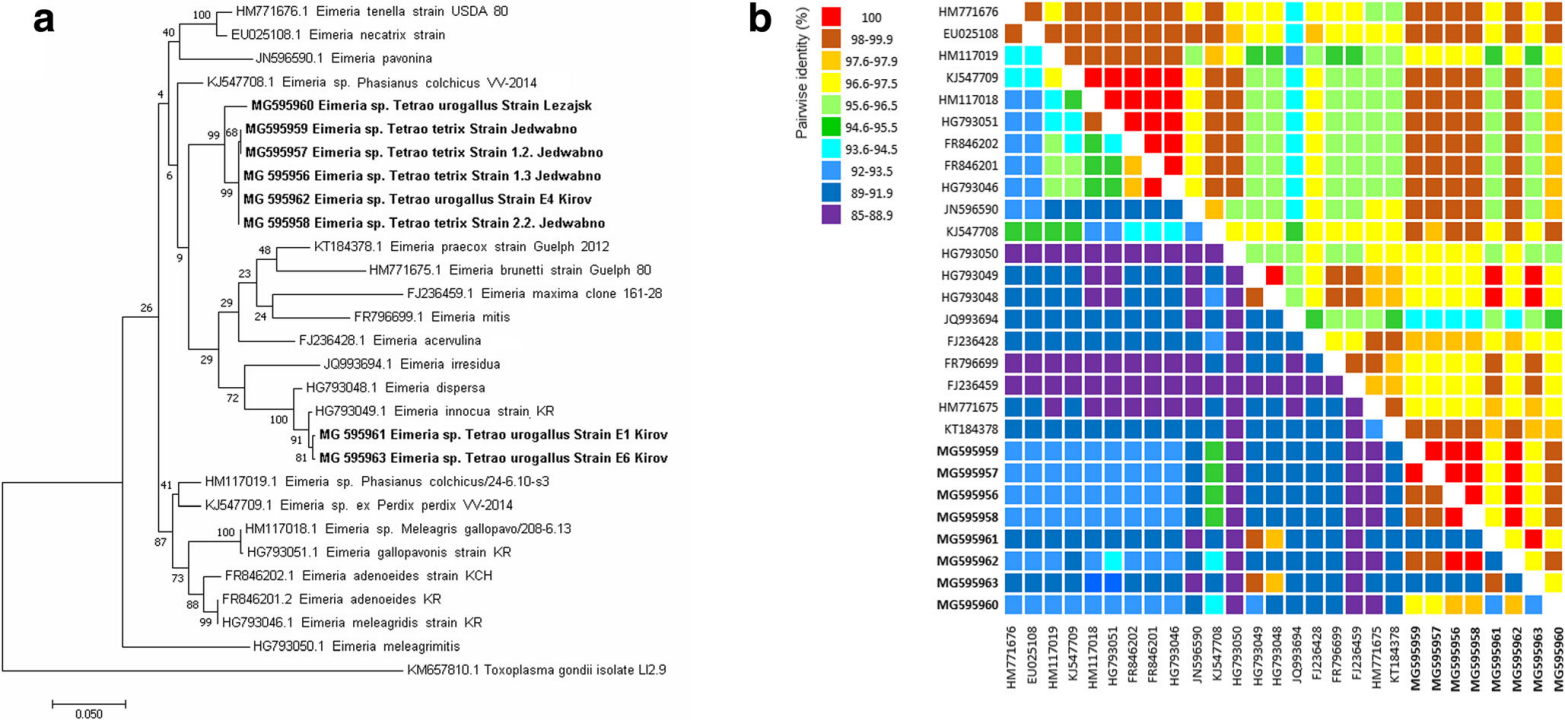
**a** The maximum likelihood phylogenetic tree of 20 cox-1 gene nucleotide sequences of various *Eimeria* species available in the GenBank database and *Eimeria* isolates from investigated grouse species obtained in this study. The tree with the highest log likelihood (− 4443.89) is shown. The percentage of trees in which the associated taxa clustered together is shown next to the branches. Initial tree(s) for the heuristic search were obtained automatically by applying Neighbor-Join and BioNJ algorithms to a matrix of pairwise distances estimated using the maximum composite likelihood (MCL) approach, and then selecting the topology with superior log likelihood value. The tree is drawn to scale, with branch lengths measured in the number of substitutions per site. All positions containing gaps and missing data were eliminated. **b** Pairwise identity matrices calculated with the Clustal W method comparing cox-1 gene sequences of 20 *Eimeria* species from the GenBank database, and *Eimeria* isolates from capercaillie and black grouse obtained in this study with the percentage of nucleotide identity of cox-1 gene (bottom left) and the percentage of amino acids in protein encoded by this gene (top right)

The phylogenetic analysis of a cox-1 gene fragment revealed that in the context of this gene, the analyzed *Eimeria* sequences belonged to two species. The first one was represented by coccidia isolated from both grouse (capercaillie: Polish strain from Leżajsk State Forestry District and strain E4 Kirov, black grouse: all strains). *Eimeria* isolated from these species are clearly different from other known coccidia isolated from the gallinaceous birds (Fig. 2a). The sequences classified in these species were characterized by high homology (99.9–100%) except the sequence belonging to the Leżajsk strain (97.5–97.6%). The other two isolates obtained from capercaillies imported from the Kirov region were similar to turkey coccidia: *E. innocua* and *E. dispersa* (97.9–99.4%) and were homologous at 99.7% to each other (Fig. 2a).

**Fig. 3:**
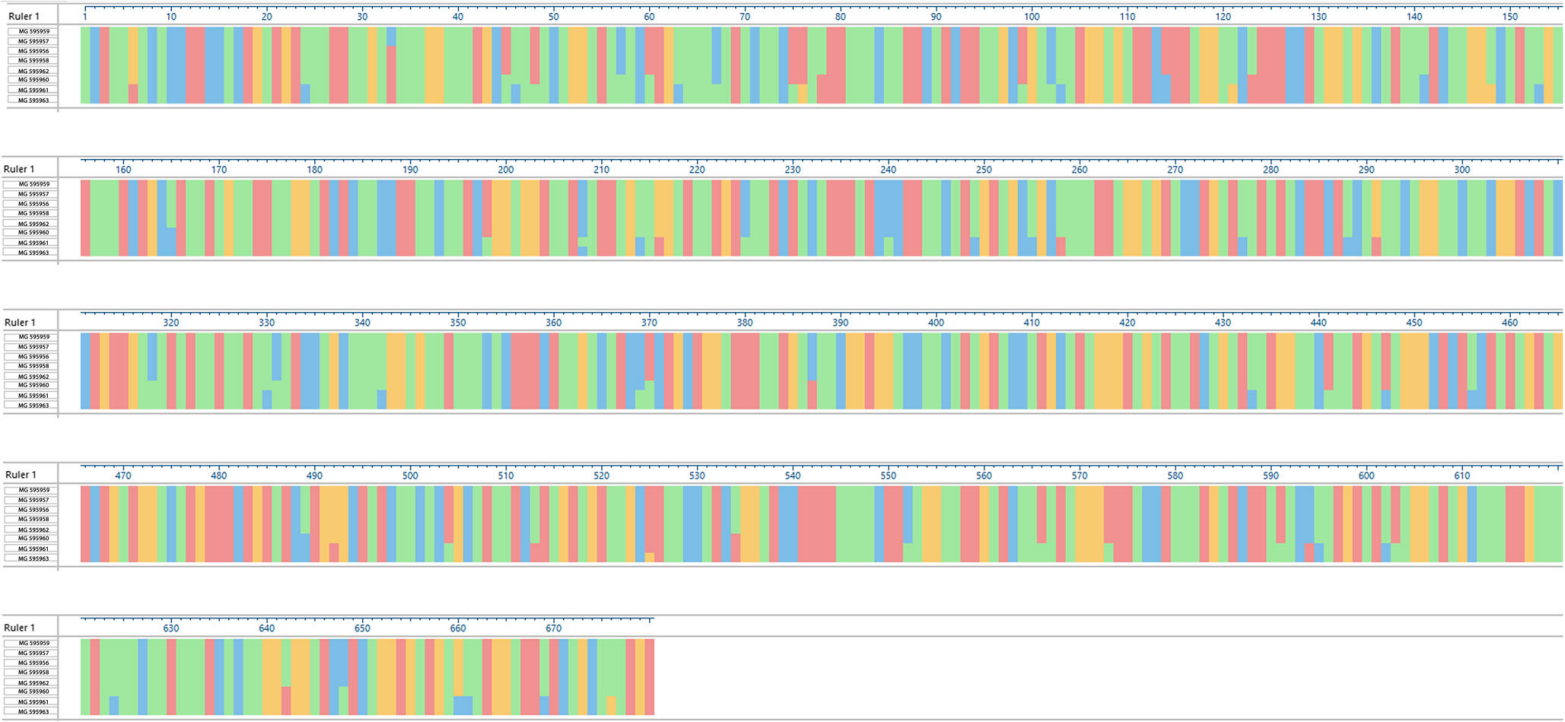
Nucleotide substitutions in the analyzed cox-1 gene fragment among the revealed isolates of *Eimeria* spp. The alignment was created with CLUSTAL W in MegAlign software (DNASTAR, USA). The following background colors indicate the following nucleotides: red—adenine (A), blue—cytosine (C), orange—guanine (G), green—thymine (T). The positions where nucleotide substitution occurred are indicated by background color change

The average nucleotide identity of the cox-1 gene between *Eimeria* isolated from grouse and other gallinaceous birds was 91.3%, whereas the average nucleotide identity calculated only between grouse strains was higher and reached 95.5% (Fig. 2b). Numerous point mutations were found in the analyzed cox-1 gene fragment. Most of them were present in Kirov strains E1 (MG 595961) and E6 (MG 595963), where a total of 45 nucleotide substitutions were revealed in comparison to the other sequences. The most common substitutions included thymine instead of cytosine (T > C, 19 mutations), adenine instead of thymine (A > T, 9 mutations), and cytosine instead of thymine (C > T, 6 mutations). In the case of Leżajsk strain (MG 595960), 16 nucleotide substitutions common for E1 and E6 Kirov strains were found, whereas in Jedwabno (MG 595959) and 1.2 Jedwabno (MG 595957) strains, only one substitution was revealed (C > A) (Fig. 3). The average identity of amino-acid sequences obtained after virtual translation was higher and reached 97.4% for all compared *Eimeria* sequences and in average 98.6% of grouse sequences (Fig. 2b).

**Fig. 4:**
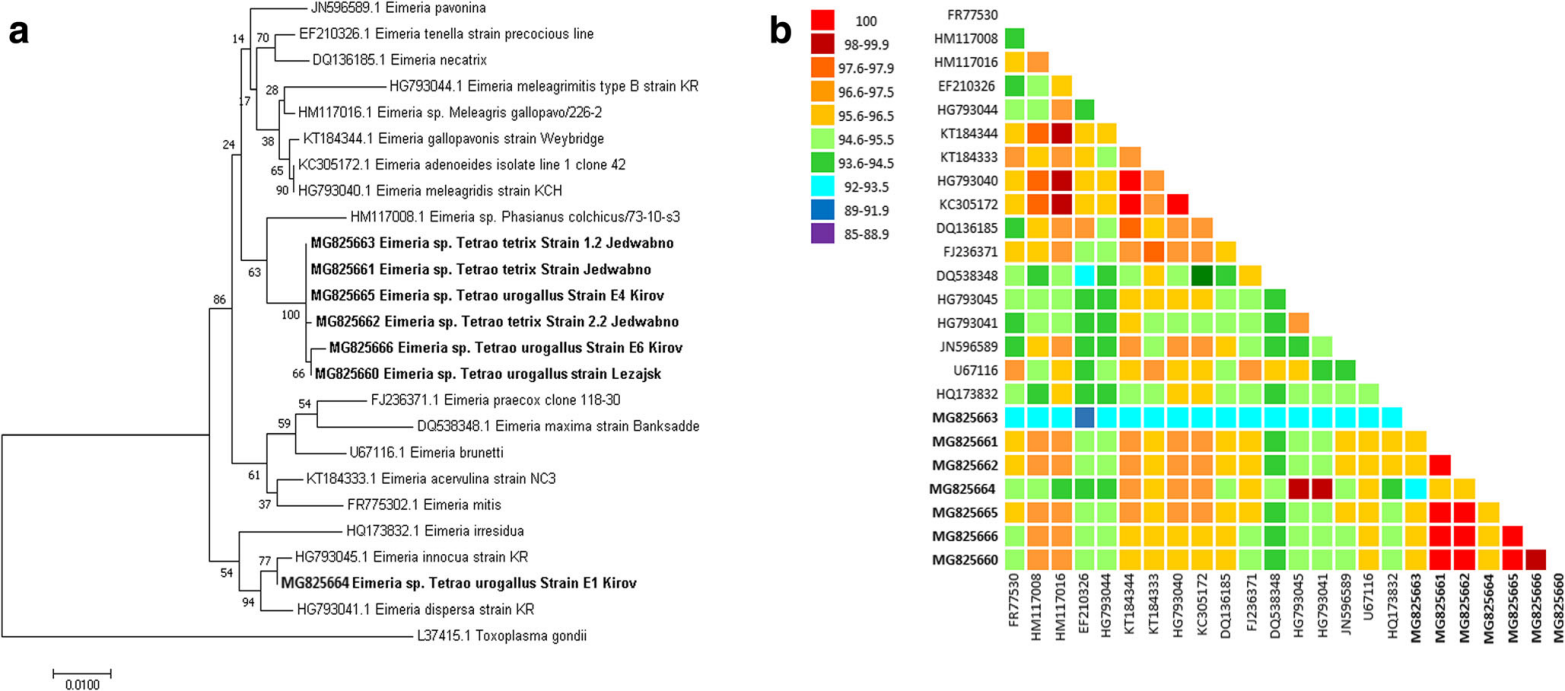
**a** The Maximum likelihood phylogenetic tree of 17 18S rRNA nucleotide sequences of various *Eimeria* species available in the GenBank database and seven *Eimeria* isolates from capercaillie and black grouse obtained in this study. The tree with the highest log likelihood (− 3697.28) is shown. The percentage of trees in which the associated taxa clustered together is shown next to the branches. Initial tree(s) for the heuristic search were obtained automatically by applying Neighbor-Join and BioNJ algorithms to a matrix of pairwise distances estimated using the maximum composite likelihood (MCL) approach, and then selecting the topology with superior log likelihood value. The tree is drawn to scale, with branch lengths measured in the number of substitutions per site. All positions containing gaps and missing data were eliminated. **b** Pairwise identity matrix calculated with the Clustal V method comparing 18S rRNA gene sequences of 17 *Eimeria* species from the GenBank database, and *Eimeria* isolates from investigated grouse species obtained in this study

**Fig. 5:**
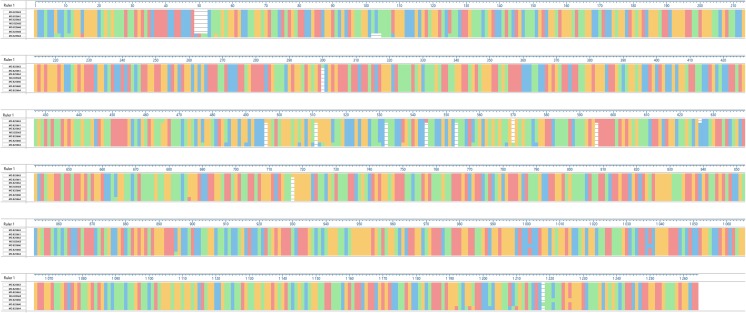
Mutations in the analyzed 18S rRNA gene fragment among the revealed isolates of *Eimeria* spp. The alignment was created with CLUSTAL W in MegAlign software (DNASTAR, USA). The following background colors indicate the following nucleotides: red—adenine (A), blue—cytosine (C), orange—guanine (G), green—thymine (T). The positions where nucleotide substitution occurred are indicated by background color change, whereas white color indicates deletion of a single nucleotide or a motif in the aligned sequences. Insertion of a motif or a single nucleotide in a single sequence results in the virtual deletion in the other sequences after alignment

The phylogenetic analysis of obtained fragment of the 18S rRNA gene sequences revealed that most of the isolates belonged to one species (including isolate E6 Kirov) similar to the pheasant strain (96.3% homology) except for the E1 Kirov isolate which belonged to other species and highly homologous to turkeys coccidia *E. dispersa* and *E. innocua* (98–99% homology). The average nucleotide identity of all analyzed sequences of this gene was 95.6%, and the average nucleotide identity between the grouse sequences only was higher and reached 97.5% (Fig. 4). The highest number of point mutations in the analyzed fragment of the 18S rRNA gene was found in E1 Kirov strain (MG 825664), in comparison to the other strains, where a total of 29 nucleotide substitutions were noted, of which C > T (8 mutations) and T > C (6 mutations) were the most common ones. An insertion of “CATT” (position 49–52 of the alignment) motif and single thymine (pos. 570 of the alignment) and a deletion of “TCT” motif in position 102–104 of the alignment were observed as well. Numerous single-nucleotide insertions were noted in the case of 2.2 Jedwabno strain (MG 8255662) where four thymine (pos. 300, 532, 544, and 553 of the alignment), three guanine (pos. 496, 511, 717 of the alignment), and one adenine insertion in pos. 595 of the alignment were found. Also, a deletion of single cytosine in pos. 626 of the alignment was observed. In the case of Leżajsk strain (MG 8255660), an insertion of single thymine in pos. 1218 of the alignment was found, whereas in E6 Kirov strain (MG 8255666), only four nucleotide substitutions were noted (Fig. 5).

## Discussion

Our investigation has revealed that coccidia are highly prevalent in both grouse species, which is consistent with literature data (Jankovska et al. 2012; Millán et al. 2008; Obeso et al. 2000). However, the prevalence of coccidia in our study was much higher than in literature, which is probably due to the fact that the published data originated from investigations performed on wild grouse and not on birds kept in captivity. The high prevalence of *Eimeria* parasites in capercaillies transported from the Kirov region could be connected with bird exposure to stress during quarantine and transport, which could lead to their suppressed immunity. On the other hand, keeping animals in confined space promotes development of parasitic invasions, especially those with the simple life cycle. This could be the reason for high coccidia invasion in the investigated black grouse samples obtained from birds from one aviary. Little is known about *Eimeria* species occurring in grouse and their life cycle. However, the high prevalence of invasions in birds kept in captivity for reintroduction purposes suggests that they could lead to serious health problems resulting in interference of the reintroduction process. The high intensity of parasitic invasions has been shown to negatively influence a population by reducing clutch size, hatching success, and survival of young birds (Isomursu et al. 2012; Watson 2013).

Literature data suggest that most prevalent coccidia species occurring in black grouse is *Eimeria lyruri* (Jankowska et al. 2012). The coccidial sequences classified in our study as a separate clade (Figs. 2 and 4) probably belong to this *Eimeria* species, but we are unable to confirm this hypothesis because none of grouse *Eimeria* sequences is available in the GenBank database. There is also data about various coccidia species prevailing in capercaillie and black grouse including *E. nadsoni*, *E. procera*, and *E. tetricis*, but description of those species is scant and the data is very old and possibly out of date (Haase 1939; Pav and Zajicek 1974).

Our investigation demonstrated that the molecular method for coccidia detection in fecal samples was much less sensitive than the traditional microscopic examination of the flotation fluid, because only 8 of the 49 samples positive in microscopy evaluation were positive in the cox-1 assay and only 7 were positive in the 18S rRNA assay. In fact, only the samples in which oocyst count was high proved to be useful for PCR assays and further molecular analyses. The above could be explained by difficulties in DNA extraction from oocysts caused by their robust wall or by insufficient disruption of oocysts. Similar observations were made by other authors (Honma et al. 2011; Bertram et al. 2015).

The parasitological screening revealed that two morphotypes of eimeriid oocysts were present in the analyzed fecal samples. This suggests invasion of two different species of coccidia in both grouse species. The newest data presented by Ogedengbe et al. (2018) suggests that the morphology-based classification could be insufficient for proper *Eimeria* classification, and that molecular analysis is more reliable in this respect. Sequencing of small genome regions, such as mitochondrial cytochrome oxidase subunit 1 (cox-1) locus and 18S rRNA gene, has been commonly used to infer the relatedness of field *Eimeria* isolates (Clark et al. 2017). The phylogenetic analysis of cox-1 gene fragment performed with the maximum likelihood suggests two coccidia species to occur in capercaillie and black grouse what is convergent with parasitological examination. One of them is characteristic only for investigated grouse species and is related (approximately 94%) to the *Eimeria* spp*.* strain isolated from ring-necked pheasants (acc no. KJ547708) (Fig. 2b). This species was the most prevalent (6 of 8 sequences) and was present in capercaillie from the Kirov region and from Leżajsk State Forestry District, and also in black grouse from the Jedwabno State Forestry District. It is well known that each coccidia species is highly specific to the host species, but our results suggest that this *Eimeria* species could occur in both capercaillie and the black grouse. We have also shown that there is no correlation between host species, host geographical origin, and nucleotide sequence of the analyzed gene fragment. The second species includes coccidia related to *E. innocua* and *E. dispersa* occurring in turkeys (approximately 98% homology) (Fig. 2b).

The phylogenetic analysis of the obtained partial 18S rRNA sequences showed a slight difference in classification of *Eimeria* spp*.* Kirov E6 strain in comparison to cox-1 gene fragment analysis, and this result is consistent with findings reported by other authors (Miska et al. 2010; Hauck and Hafez 2012). The above differences could result from various extents of mutation and recombination processes in both of the analyzed genes (Miska et al. 2010). Nevertheless, some similarities between both analyzed genes were observed. Most of the sequences obtained from investigated grouse coccidia belonged to one species partially homologous to the *Eimeria* isolated from ring-necked pheasant (approx. 96% for 18S rRNA genes) while E1 Kirov strain is related to *E. innocua* and *E. dispersa* (98–99% homology). The only difference is classification of the E6 Kirov strain obtained from capercaillie in the analyses of both gene sequences. The 18S rRNA gene is more conservative than cox-1 gene is (the average homology of all analyzed sequences was 95.6 and 91.3%, respectively) (Figs. 2b and 3b). Compared to the 18S rRNA gene, the cox-1 gene is quickly evolving (sequence divergence rate of 1–2% per million years) and for this reason, the cox-1 gene sequences are a better base for the identification and phylogenetic analysis than the 18S rRNA gene sequences (El-Sherry et al. 2013; Ogedengbe et al. 2018). Summarizing, the phylogenetic tree based on mitochondrial gene sequences is in this case more reliable.

To conclude, this paper constitutes the first report of the molecular characterization of an *Eimeria* species isolated from capercaillie and black grouse. The morphological and molecular analyses suggest that two species of coccidia could be present in both black grouse and capercaillie. From the reason of limited knowledge, further investigations are needed with the use of coupled parasitological and molecular analyses.
